# Creutzfeldt-Jakob Disease Presenting as Nonconvulsive Status Epilepticus

**DOI:** 10.1155/2018/3092018

**Published:** 2018-11-04

**Authors:** Aleksei Rakitin, Riina Vibo, Vaiko Veikat, Anne Õunapuu, Aive Liigant, Sulev Haldre

**Affiliations:** ^1^Department of Neurology and Neurosurgery, University of Tartu, Estonia; ^2^Department of Psychiatry, University of Tartu, Estonia

## Abstract

Creutzfeldt-Jakob disease is a rare, rapidly progressive spongiform encephalopathy in humans. EEG plays an important role in diagnosing this disease. In some patients, epileptic activity and encephalopathy from various aetiologies may share morphological features on EEG. This similarity could create difficulties in EEG interpretation, especially if the patient presents with disturbed consciousness. In this case report, a 74-year-old female with Creutzfeldt-Jakob disease presented initially with rapidly progressive impairment of consciousness and focal epileptiform activity on EEG. An EEG performed 25 days later showed periodic sharp-wave complexes with triphasic morphology at a rate of 0.5 Hz, compatible with a diagnosis of Creutzfeldt-Jakob disease. Based on these results, we recommend that a diagnosis of Creutzfeldt-Jakob disease be considered in patients presenting with a rapid deterioration of consciousness and a clinical presentation of nonconvulsive status epilepticus. Monitoring these patients with serial EEGs could be useful to establish an accurate diagnosis.

## 1. Introduction

EEG is required for a diagnosis of nonconvulsive status epilepticus (NCSE) in comatose patients [1]. In many cases, it may be difficult to distinguish seizure activity in the EEG from nonepileptic (metabolic, postanoxic, or spongiform) EEG features in patients with confusion [2]. In an acute clinical situation, this differentiation is critical because it determines the aggressiveness of antiepileptic drug treatment. For these reasons, diagnosing NCSE remains a challenge for most clinicians. To solve this issue, Beniczky et al. recently proposed unified clinical and EEG criteria for the diagnosis of NCSE [3]. However, interpreting periodic abnormalities in patients with impaired consciousness is complicated, particularly when epileptiform activity and encephalopathy coexist. Here, we describe the case of an elderly woman with sporadic Creutzfeldt-Jakob disease (sCJD) who, shortly after presentation of the initial symptoms of the disease, demonstrated a rapid deterioration of consciousness and EEG features suggestive of NCSE.

## 2. Case Report

A 74-year-old female patient was referred to the Tartu University Clinic with impaired consciousness and tetraparesis from a local hospital where she had been diagnosed with an ischaemic stroke. A month before her presentation to our clinic, the patient's symptoms had appeared as only clumsiness and a slight impairment of the sensations in her left hand. At home, her clinical state progressively worsened, and she developed difficulty with speech, walking, and swallowing. Mental deterioration of the patient was not reported, possibly due to her rapid impairment of consciousness. The patient's medical history included a myocardial infarction in 2008, hypothyreosis, increased blood pressure, and tension headaches. However, the patient was completely independent and lived with her husband.

Upon arrival to the Tartu University Clinic, the patient exhibited disturbed consciousness with a Glasgow Coma Scale (GCS) score of 9 points. Laboratory tests revealed leucocytosis and increased C-reactive protein levels. She was diagnosed with and treated for right-sided pneumonia. T1, T2, FLAIR with gadolinium contrast medium and diffusion-weighted brain MRI was performed, showing only small periventricular and subcortical white matter lesions compatible with ischaemic leukoencephalopathy. The patient was not diagnosed with any infectious, inflammatory, or neoplastic aetiology. Lumbar puncture revealed an absence of white blood cells and a protein concentration of 0.28 g/L.

**Figure 1:**
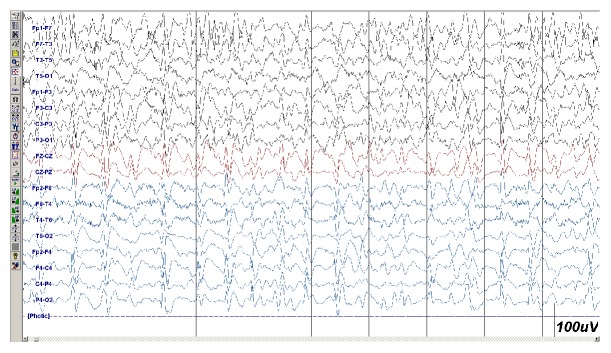
The initial EEG showed pseudoperiodic lateralized epileptiform discharges over the right hemisphere with a frequency of 2–3 Hz. Calibration: 1 s between vertical lines and 100 *µ*V per vertical unit. Low filter: 1 Hz; high filter: 35 Hz; notch filter: 50 Hz.

On the day of admission, a 20-minute standard EEG was obtained. During the examination, the patient's GCS score was 9 points, and she exhibited occasional, subtle clonic jerks in her left arm. The EEG showed pseudoperiodic lateralized epileptiform discharges (PLEDs) over the right hemisphere at a frequency of 2–3 Hz (Figure 1), which were time-locked with the clinical motor signs (video sequence 1). Surface voltage mapping showed a dipole configuration with maximal negativity over the right frontoparietal region. The nociceptive stimulation was performed, without affecting PLEDs.

After the administration of intravenous diazepam (5 mg), the epileptiform activity and left arm jerks were completely resolved, and the EEG exhibited diffuse delta activity with a frequency of 1.5 Hz. However, the patient's mental state did not improve. Based on these findings, the patient was diagnosed with possible NCSE, and antiepileptic treatment with carbamazepine and intravenous valproate was initiated. The patient did not respond with clinical improvement, despite therapeutic plasma levels of the anticonvulsive medications and the introduction of propofol anaesthesia in the intensive care unit.

**Figure 2:**
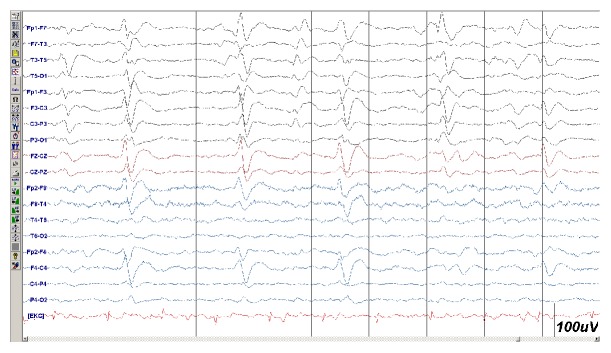
The second EEG performed 25 days after the initial EEG showed severe slowing of background activity and periodic sharp-wave complexes with triphasic morphology at a rate of 0.5 Hz. Calibration: 1 s between vertical lines and 100 *µ*V per vertical unit. Low filter: 1 Hz; high filter: 35 Hz; notch filter: 50 Hz.

A brain biopsy was performed on the right parietal lobe, revealing pathological results compatible with the diagnosis of sporadic CJD. The CSF of the patient was positive for the 14-3-3 brain protein. The patient had an intracerebral haemorrhage in the right hemisphere as a complication of the brain biopsy, and the haematoma was surgically removed.  Figure 2 shows EEG results performed 25 days after the first EEG. The exam revealed a severe slowing of the background activity and periodic sharp-wave complexes (PSWCs) with triphasic morphology at a rate of 0.5 Hz. She was stable after the operation but died 34 days after admission. The patient's total disease duration was 60 days.

## 3. Discussion

This case highlights an unusual presentation of sCJD characterised by a rapidly progressive impairment of consciousness and seizure activity on EEG, leading to a diagnosis of NCSE. Epileptic seizures occur in approximately 15% of patients with sCJD and typically present late in the disease [4]. However, seizures are uncommon as a presenting symptom of sCJD and occur in only 3% of cases [5].

The current literature highlights an interesting discussion regarding whether the EEG results suggestive of NCSE in those patients represent real seizure activity or whether the epileptiform discharges are misinterpreted and instead represent nonepileptiform PSWCs typical of CJD [2, 6]. Indeed, triphasic waves (TWs) and epileptiform transients during NCSE share morphological features that may create diagnostic ambiguity. Boulanger et al. showed that NCSE-associated epileptiform discharges exhibited a higher frequency (2.4 versus 1.8 Hz), a shorter duration of phase one, and less generalized background slowing when compared to TWs. The lag of phase two is usually absent in NCSE, and noxious or auditory stimuli could increase the TWs while not affecting the epileptiform patterns [7]. Epileptic discharges are usually time-locked with the clinical motor signs; however, myocloni related to the CJD may occur before, after, and during the PSWCs [8]. The effects of drugs such as benzodiazepines on the EEG could be similar under both conditions, with the attenuation or abolishment of TWs and epileptiform discharges [9]. In NCSE, the EEG changes could be accompanied by marked clinical improvement; however, the absence of clinical improvement does not exclude an epileptic condition [1].

In this case, several NCSE-compatible clinical and electrophysiological anomalies were present: severely disturbed consciousness, periodically occurring subtle clonic jerks in the left arm, time-locked with the PLEDs in the right hemisphere, and a positive effect of benzodiazepine on EEG and motor phenomena. However, the mental state of the patient did not resolve. This discrepancy may be explained by the fact that the patient suffered from CJD and had already presented an abnormal basal mental status. This patient could also have been diagnosed with NCSE according to recently published EEG criteria for NCSE, including the presence of epileptiform discharges with a frequency that exceeded 2.5 Hz and subtle clinical phenomena present during those discharges [3].

However, clonic jerks time-locked with the contralateral PLEDs could be interpreted as epilepsia partialis continua (EPC). As reviewed by Taskiran et al., few CJD cases associated with EPC have been reported in the literature thus far [10]. Moreover, in all of these cases, no disturbance of consciousness has been reported. Indeed, EPC by definition includes spontaneous regular or irregular clonic muscular twitching that affects a limited part of the body, usually with no disturbance of consciousness [11]. The severely impaired consciousness in our patient, shortly after the onset of disease, in the absence of any structural lesion on brain MRI supports the NCSE diagnosis. Hyperintensities in basal ganglia are highly suggestive of sCJD, whereas cortical hyperintensities could be present in both NCSE and sCJD [12]. However, the absence of hyperintensities in the basal ganglia and cortical areas on brain MRI sequences in pathologically proven CJD cases is also reported earlier, making correct diagnosis in our case more difficult [2, 13]. Detection of 14-3-3 protein in CSF of a patient has only a moderate diagnostic value for sCJD, since it may result also from extensive damage due to status epilepticus [2].

The second EEG of the patient (Figure 2) showed a PSWC at a rate of 0.5 Hz with a slow background; this EEG suggested a diagnosis of CJD and was clearly different from the first EEG (Figure 1). This finding suggests that it would be reasonable to withdraw anticonvulsant treatment at this stage. Earlier reports showed that the mean survival time of patients with CJD is 8.2 months (range: 1.5–58 months) [4]. The relatively fast disease course in the patient presented here suggests the negative impact of seizure activity on the survival time. Aggressive treatment and complication of brain biopsy may also play a role in rapid evolution of this case.

In summary, the interpretation of EEG and clinical features in early stage CJD patients can be difficult. This case report confirms earlier findings that a diagnosis of CJD should be considered in patients presenting with a rapid deterioration of consciousness and a clinical picture of NCSE. However, a diagnosis of NCSE should be made using strict clinical and EEG criteria. Monitoring these patients with serial EEGs could be useful to establish an accurate diagnosis.

## Supplementary Materials

Supplementary Materials Video 1. EEG showed pseudoperiodic lateralized epileptiform discharges over right hemisphere with a phase reversal at the C4 and P4 electrodes and a frequency of 1–2 Hz. The patient had clonic jerks in the left arm that were time-locked with sharp waves. Calibration: 1 s between vertical lines and 100 *µ*V per vertical unit. Low filter: 1 Hz; high filter: 35 Hz; notch filter: 50 Hz.
